# Global land use implications of dietary trends

**DOI:** 10.1371/journal.pone.0200781

**Published:** 2018-08-08

**Authors:** Sarah Rizvi, Chris Pagnutti, Evan Fraser, Chris T. Bauch, Madhur Anand

**Affiliations:** 1 School of Environmental Science, University of Guelph, Guelph, Ontario, Canada; 2 Department of Geography, University of Guelph, Guelph, Ontario, Canada; 3 Department of Applied Mathematics, University of Waterloo, Waterloo, Ontario, Canada; Michigan State University, UNITED STATES

## Abstract

Global food security and agricultural land management represent two urgent and intimately related challenges that humans must face. We quantify the changes in the global agricultural land footprint if the world were to adhere to the dietary guidelines put forth by the United States Department of Agriculture (USDA), while accounting for the land use change incurred by import/export required to meet those guidelines. We analyze data at country, continental, and global levels. USDA guidelines are viewed as an improvement on the current land-intensive diet of the average American, but despite this our results show that global adherence to the guidelines would require 1 gigahectare of additional land—roughly the size of Canada—under current agricultural practice. The results also show a strong divide between Eastern and Western hemispheres, with many Western hemisphere countries showing net land sparing under a USDA guideline diet, while many Eastern hemisphere countries show net land use increase under a USDA guideline diet. We conclude that national dietary guidelines should be developed using not just health but also global land use and equity as criteria. Because global lands are a limited resource, national dietary guidelines also need to be coordinated internationally, in much the same way greenhouse gas emissions are increasingly coordinated.

## Introduction

Increasing pressures on land and other natural resources such as water is largely attributed to the increase in demand for agricultural products [1]. The agricultural sector is extremely resource-intensive and continues to transform itself as populations grow. Global food production is the largest user of fresh water and uses approximately 38% of the land on Earth [1,2]. An estimated 62% of the remaining global land surface is either unsuitable for cultivation on account of soil, climate topography, or urban development (30%) or is covered in natural land states like forests (32%), so very little land is available for agricultural expansion that does not destroy native land states. Hence, more efficient agricultural production is urgently needed [3].

However, approximately 12% of the world remains undernourished [2]. According to estimates from the Food and Agriculture Organization of the United Nations (FAO), the world will need to produce 70% more food by 2050 to meet increased demand [3]. The global food system is at a point of change where a thorough understanding of the relationship between food consumption patterns, agricultural production and distribution is required to improve the overall sustainability of the system [4]. It has become important now more than ever to make global agricultural production both sustainable and equitable.

The global distribution of diet may play a major role in achieving this goal. Food consumption patterns vary widely between countries. Average caloric intake in least developed, developing, and industrialised countries varies widely; 2,120, 2,640, and 3,430 kcal per person per day, respectively [5,6]. In many developing countries the average intake is even lower than 2,120 kcal per person per day, resulting in undernourishment [3].

National dietary guidelines provide guidance on what constitutes a healthy diet, especially in industrialised countries where individuals have access to a wide choice of foods. The United States Department of Agriculture (USDA) released *The Dietary Guidelines for Americans*, *2010* (“USDA guidelines” hereafter) to promote a healthy diet low in calories and saturated fats. The dietary guidelines are divided by food groups and daily caloric intake levels depending on age, sex, and physiological status (Table 1) [7]. Comparing the recommended food group servings to current agricultural outputs and dietary practice reported in food balance sheets from the FAO—both in the United States and many other industrialised countries—shows a mismatch between current and guideline diets. For instance, in North America, the consumption of land-intensive foods like meat is higher than the USDA guidelines recommend, and consumption of land-sparing foods like vegetables is too low [8,9].

**Table 1:** Daily recommended caloric intake of each food group as outlined by the United States Department of Agriculture Food Guide. Table adapted from the USDA *Dietary Guidelines for Americans 2010* [7]. Food groups are divided into 6 categories with servings determined by caloric levels. The caloric levels are assigned based on sex, physiological status and age.

					Daily Calorie Level							
Food Group	1000	1200	1400	1600	1800	2000	2200	2400	2600	2800	3000	3200
					(Servings)							
**Fruit (Cups)**	1.0	1.0	1.5	1.5	1.5	2.0	2.0	2.0	2.0	2.5	2.5	2.5
**Vegetables (Cups)**	1.0	1.5	1.5	2.0	2.5	2.5	3.0	3.0	3.5	3.5	4.0	4.0
**Grains**	3.0	4.0	5.0	5.0	6.0	6.0	7.0	8.0	9.0	10.0	10.0	10.0
**Whole-grain portion (oz-eq)**	1.5	2.0	2.5	3.0	3.0	3.0	3.5	4.0	4.5	5.0	5.0	5.0
**Meat and Beans (oz-eq)**	2.0	3.0	4.0	5.0	5.0	5.5	6.0	6.5	6.5	7.0	7.0	7.0
**Milk (cups)**	2.0	2.0	2.0	3.0	3.0	3.0	3.0	3.0	3.0	3.0	3.0	3.0
**Oils (tsp)**	3.0	4.0	4.0	5.0	5.0	6.0	6.0	7.0	8.0	8.0	10.0	10.0
**Discretionary calorie allowance**	165	171	171	132	195	267	290	362	410	426	512	648

It is well known that there is not enough land for land-intensive diets such as those currently practiced in the United States to be applied globally [3]. However, it is not known whether the healthier and less land-intensive diets such as described in the USDA guidelines would have the same limitation. This could result in net land sparing attributable to countries such as the United States where meat consumption declines under a USDA guideline diet. At the same time, land use attributable to the poorest countries would increase, as individuals gain the calories required to avoid malnourishment. This would clearly make global diets more equitable, but it is not clear what the net effect on land use would be.

Therefore, in this paper we build on the global land use change literature, which explores both the drivers and consequences of how human decisions affect landscapes [10,11], to address the question: Is there enough land worldwide under current agricultural practice for every country to adhere to the USDA guidelines?

## Methods

We used the USDA guidelines because they are comprehensive and well articulated (Table 1) [7]. Also, many lower-income countries are beginning to adopt a more westernized lifestyle including a diet similar to that expressed in the USDA guidelines, so the study is consistent with ongoing global dietary trends.

We used the FAOSTAT database [2] to compile the food supply quantity for each of the commodity aggregates listed in Table 1 and grouped them according to the major food groups recognized in the USDA MyPyramid model: fruits, vegetables, grains, meat/protein, dairy, oils and discretional [7]. For beverages, oils, sugar, butter and stimulants we converted the processed quantities to equivalent primary quantities (e.g. wine to grapes, beer to barley, butter to milk etc.) using conversion factors given by the FAO [12]. The food supply quantity derived from the domestic supply and reported in the Food Balance Sheets includes production plus imports minus exports. Thus, when calculating domestic land use, we subtracted the imported quantity to determine domestic land used for growing food. We also used these data to compute the import dependency ratio, defined as the ratio of the import quantity to the domestic supply quantity, for each country and each commodity. This ratio is used at a later step of the analysis (see below).

Next we took the recommended daily serving sizes of each food group based assuming an intake of 2000 kcal/day and converted those to masses using the food balance sheets handbook given by the FAO [13]. For each country we multiplied each of these masses by 365 (days) times the population of the country to get the quantity of each food group that would be required in order for that country to adhere to the USDA guidelines in a year. A country’s surplus of each food group was taken to be the actual food supply for each food group minus the corresponding quantity that would be required to meet the USDA guidelines. A negative surplus is interpreted as a deficit, meaning that the country would need more food from that group to follow the guidelines.

For each country the surplus of each food group was divided into two parts: one that was produced within that country (domestic), and one that was produced outside of that country (displaced) according to the import dependency ratio [13]. To meet the dietary guidelines, we allow that imports may be increased, exports may be changed to domestic production, and domestic production may be expanded where possible. For example, suppose a country’s domestic supply is *X* tonnes of some commodity and it imports Y tonnes of the same commodity. The import dependency ratio is then *Y*/*X*. Now suppose that the amount of that commodity required by that country to meet the guidelines is *Z* tonnes. The surplus is given by *S* = *X*+*Y*-*Z*. We assume the surplus can be divided into two parts according to the domestic part S_d_ = S*(1-*Y*/*X*) and the imported (displaced) part *S*_i_ = S*(*Y*/*X*).

For the domestic portion of the surplus, the change in agricultural land area within that country that is required to meet the USDA guidelines was taken to be the domestic surplus *S*_d_ divided by that country’s combined yield of all commodities in the given food group (Table A in S1 Appendix) [2,7]. The change in agricultural land area outside of that country was computed in the same way, but using the displaced surplus *S*_i_ and the world average yields. Yields for crops can be found in the FAOSTAT database. For livestock products we estimated yield in terms of production per hectare of land. The details of the calculations and the corresponding Python script appear in Supporting Information (Text A and B in S1 Appendix). The code we used for the analysis is also available on Github (https://github.com/Pacopag/faolyzer).

Using this approach, we converted the USDA guidelines to land area required for the guideline diet at the level of country, continent, and world. We wished to estimate a conservative lower bound on the amount of land needed to meet the guidelines, if countries were to switch to the USDA guidelines in 2010. Hence, instead of relying on model-based projections for future demographics and possible dietary trends, we used historical FAOSTAT country-level data and estimated the amount of land required for the guideline diet, given the observed (lower) historical population sizes and agricultural activity until 2010. Hence, the resulting data point for each year represents the amount of land spared or required in that year, if the given country had been adhering to the USDA guidelines. Although we generated these estimates for 1960 to 2010 to evaluate past trends, the values for 2010 are most relevant to the current situation and generate a lower bound for possible future land requirements. Hence we focus on the 2010 estimates for our conclusions.

## Results

### Global analysis

**Fig 1:**
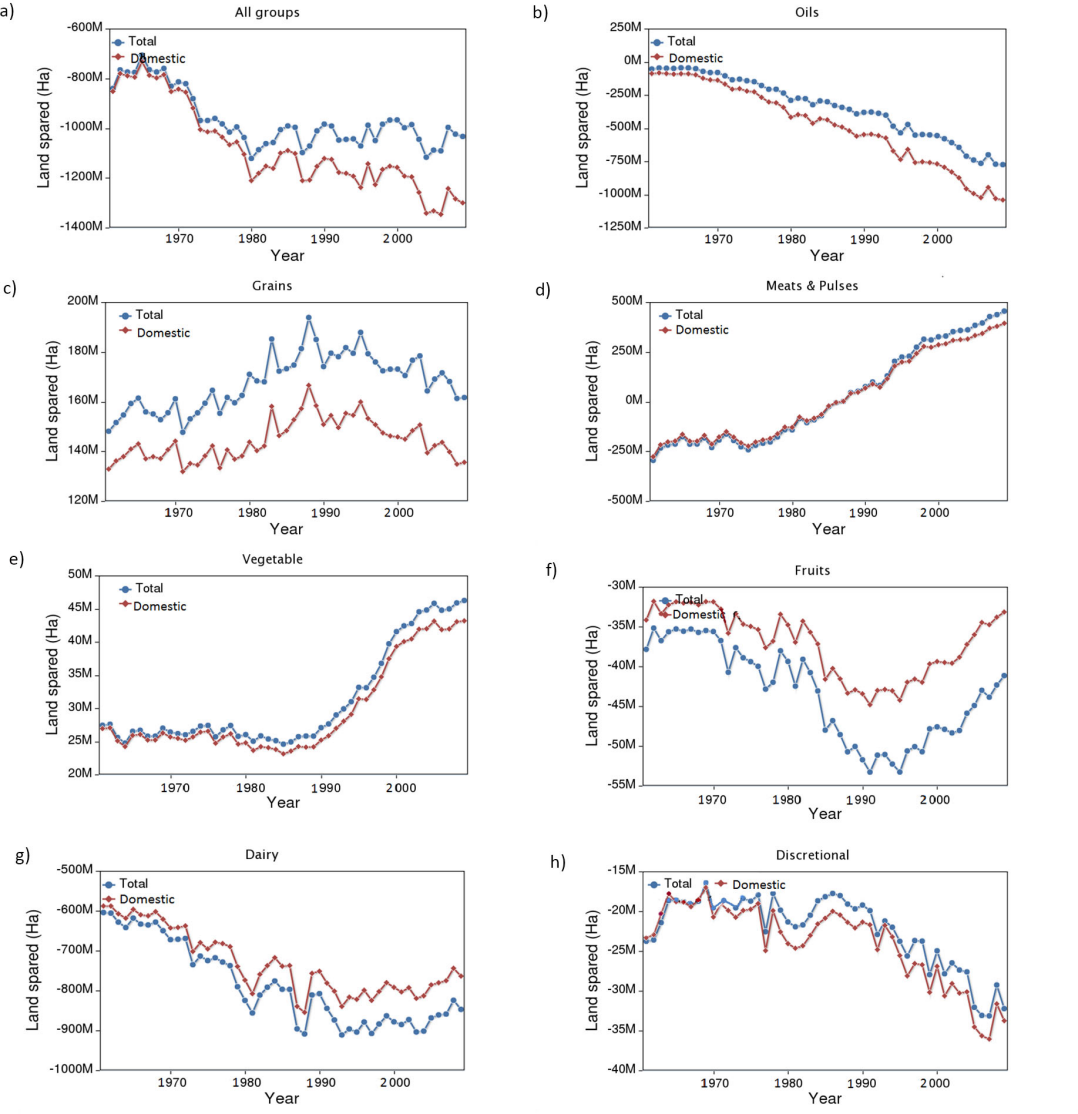
There is not enough land in the world to allow everyone to eat a USDA guideline diet. Plot shows net amount of land spared (or required) to meet the USDA *Dietary Guidelines for Americans 2010*, by year for (a) all food groups, and for (b) oils, (c) grains, (d) meat and pulses, (e) vegetables, (f) fruits, (g) dairy, and (h) discretional. Red depicts the amount of land spared or required based only on domestic production while the blue line combines domestic land and displaced land (land use a country generates elsewhere by relying on food imports) to depict a total amount of land spared (or required). A net positive value for land spared means less land would be required under a change to a USDA guideline diet, while a net negative value means more land would be required to meet the guidelines (a “land deficit”). The gap between domestic and total land spared for all groups is nonzero due to discrepancies in the FAO dataset; the two curves should match one another.

On a global scale it is apparent that certain food groups are driving most changes in agriculture. We observe that if the world were to alter its food consumption to meet the USDA guidelines, there would need to be a dramatic and unsustainable increase in agricultural lands (Fig 1).

Overall, for the world to meet the guidelines, additional land is required for fruits, dairy and oils and discretional products (Fig 1). In contrast, significant amounts of land would be spared in the meat, vegetables and grain sectors. This trend is common to most continents except Africa (Fig A in S1 Appendix). In total for all food groups, approximately 1 gigahectare (Gha) of additional land is required to meet the guidelines (Fig 1, “all groups”, 2010 data point). 1 Gha of land is roughly the size of Canada and exceeds the amount of fertile land currently available worldwide. Hence, the current USDA guidelines do not go far enough in terms of setting up a globally sustainable dietary practice.

Our analysis also shows temporal trends in land spared or required under the guidelines (Fig 1). Required land has been steadfastly increasing since 1960 (Fig 1, “all groups”) due to increasing global population.

### Analysis by continent

**Fig 2:**
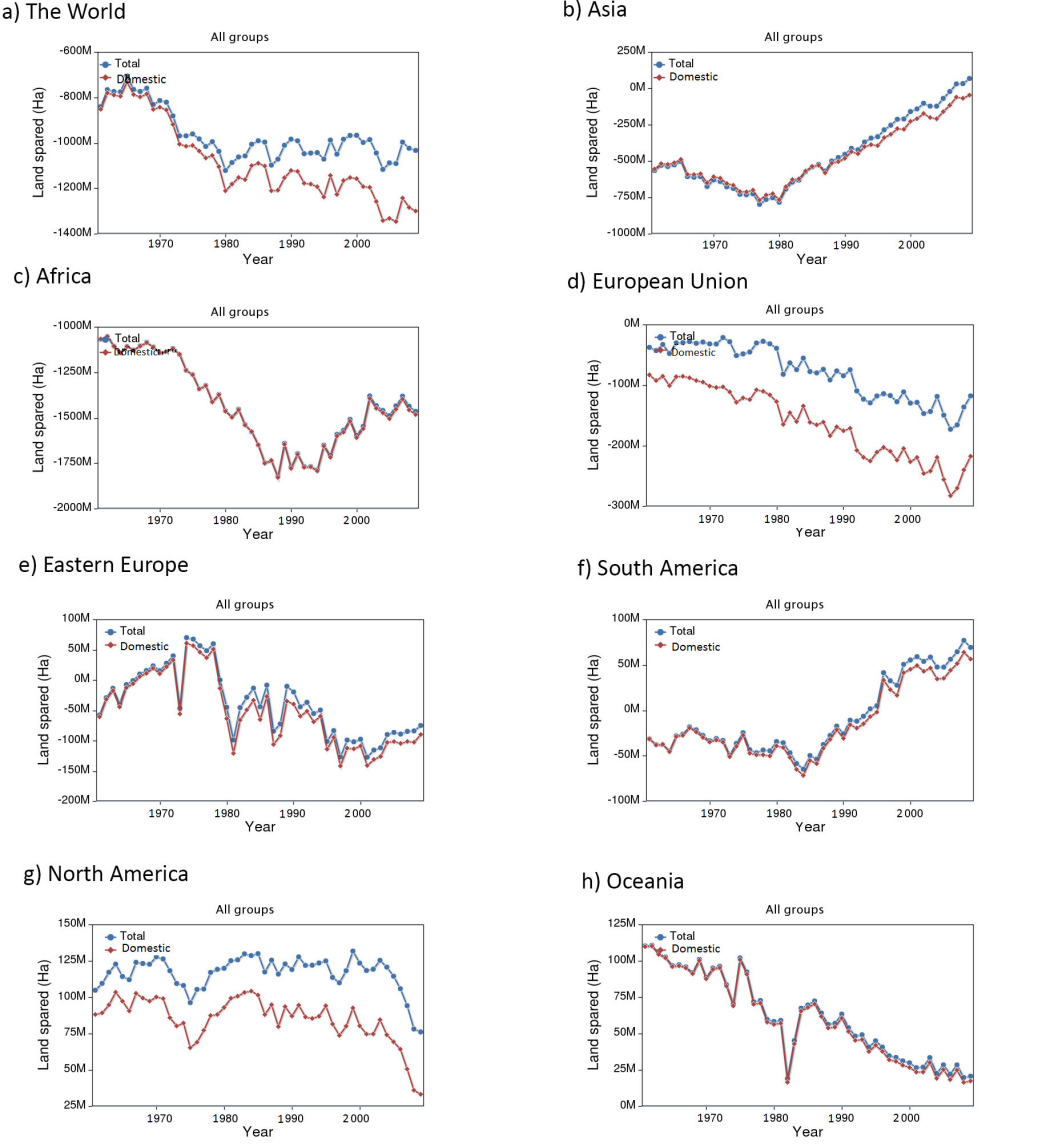
Continents differ widely in land spared (or required) under USDA guideline diet. Plot shows net amount of land spared (or required) to meet the *Dietary Guidelines* in each continent, by year for (a) the world, (b) Asia, (c) Africa, (d) European Union, (e) Eastern Europe, (f) South America, (g) North America and (h) Oceania. Red depicts the amount of land spared or required based only on domestic production while the blue line combines domestic land and displaced land (land use a country generates elsewhere by relying on food imports) to depict a total amount of land spared (or required). A net positive value for land spared means less land would be required under a change to a USDA guideline diet, while a net negative value means more land would be required to meet the guidelines (a “land deficit”).

The challenges of providing stable access to adequate food are exacerbated by inequitable dietary patterns of over- and under-consumption between countries and continents. Some of these issues become apparent when we analyze data at the continental level, at which notable common trends in consumption patterns and the associated land requirements emerge. For instance, North America and the European Union displace more land than any other continents, due to food imports (Fig 2). If North and South America shifted to USDA guidelines, they would spare a moderate amount of land from changing to a less land-intensive diet. In contrast, Africa, Eastern Europe, the European Union and Oceania would cause a large land deficit. The impact of Asia shifting to USDA guidelines would be almost neutral, although the historical trend suggests this will not be the case in the near future. The fact that the European Union (where malnourishment is currently uncommon) would cause a land deficit by shifting to the USDA guidelines suggests that the guidelines are unsustainable when it comes to land-intensive foods like meat.

For most decades, Asia would have caused a net land deficit by shifting to the USDA guidelines, since it was (and remains) a relatively under-nourished part of the world (Fig 2B). An inflection point appears in the Asian dataset in 1980, when countries like China and India began liberalizing their economies. Most notable are increases in land use for meat and grains as Asia slowly begins to adopt a more westernized diet (Fig A in S1 Appendix). This suggests that while Asia has increased land use rapidly, equity in resource distribution at the sub-continental level is imbalanced. For instance, one third of Indians are undernourished and continue to live under food insecurity [3]. Inequities in global trading and extension services as well as poor infrastructure trap populations in Asia in poverty. However, future improvements towards equal land use change may better harness agricultural yields to align the Asian diet with those of wealthier and more sustainable areas of the world, such as the European Union.

Africa would require more land to meet the guidelines than any other continent. In fact, most of the additional land required to meet the guidelines globally would be the result of dietary shifts in Africa. This is not surprising because undernourishment is widespread in Africa [14]. However, an inflection point, probably corresponding to growth in some African economies, occurs in 1990 (Fig 2C). Almost all of the additional land required to meet the guidelines would be the result of increased dairy consumption (Fig A in S1 Appendix). Although the extra land required to meet the guidelines in Africa is impossibly large (more land is needed than what is available), Africa also stands the most to gain with respect to growing agricultural yields [15]. Thus, although it is not currently possible to bring the African diet in line with that of the USA or the European Union without a net growth in agricultural lands, future improvements in agricultural practices in Africa may help to close the gap.

The European Union would also require a significant amount of land to meet the USDA guidelines. Almost all of the additional land needed would be the result of increased dairy and fruit land use, a trend common to most of Europe (Fig A in S1 Appendix). We note that displaced land (from buying food imports) contributes strongly to European Union land use, and exceeds displaced land use in North America (Fig 2D). Interestingly, the land requirements for the European Union indicate the need for more displaced lands than domestic land. This suggests that an American diet is unsustainable from a land use perspective, domestically speaking.

Land use in Eastern Europe has fluctuated significantly over time (Fig 2E). After the late 1980s, a land use deficit developed in the Eastern Europe dataset, and has largely persisted in recent years. Therefore, in order to meet the USDA guidelines, Eastern Europe would require a large amount of new land.

North America can spare a significant amount of land, should the USDA guidelines be followed. The stems largely from meat, grain and vegetable land use (Fig A in S1 Appendix) [16]. Land use for meat is greater in North America than any other continent, and as a result, land use displacement in North America is also significant (Fig 2G).

South America can also spare a significant amount of land by meeting the guidelines, mostly from land sparing due to meat and grains, followed by vegetables and discretional products. South America shows a steady increase in land use since 1984 (Fig 2F). This trend is overwhelmingly due to rapid increases in land use for meat. Thus, reducing meat consumption in South America shows strong potential for sparing land (Fig A in S1 Appendix). Finally, Oceania can spare a small amount of land if the guidelines are met, primarily from meat, grains and vegetables (Fig 2H; Fig A in S1 Appendix).

### World map

**Fig 3:**
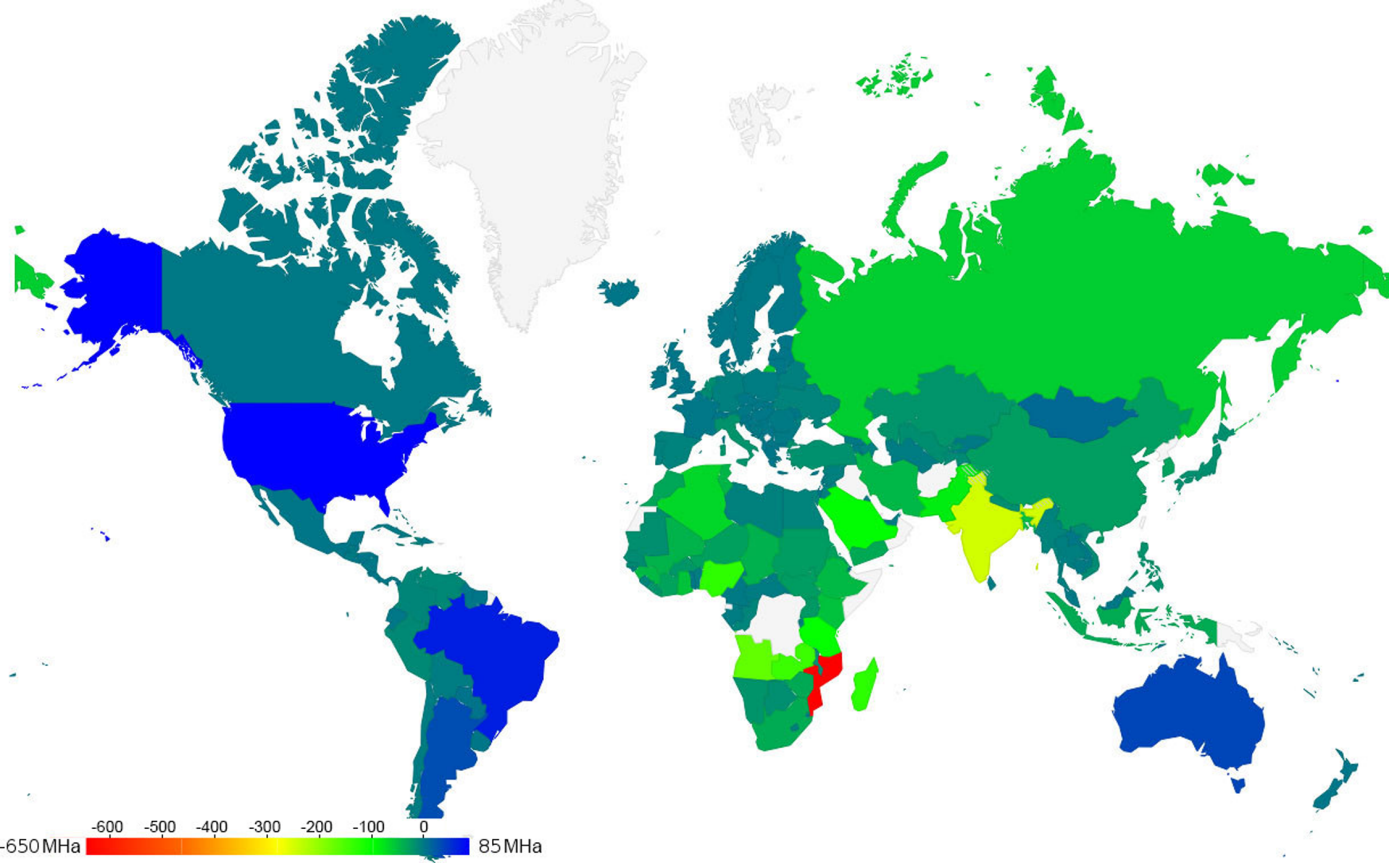
A western/eastern hemispheric divide in land spared versus land required by a USDA guideline diet. Land spared or required in 2010 by country, in millions of hectares (MHa). According to the scale, countries that would reduce global land use by changing to a USDA guideline diet (net positive land spared) are indicated in blue and teal, while countries that would require extra land to meet the guidelines (net negative land spared) are indicated in red, yellow or green. The map was created by the authors from FAOSTAT data using the Google Maps API (https://developers.google.com/maps/ with Apache License Version 2.0) [17].

We also created a world map with our results, showing net land spared or required for each country to shift to a USDA guideline diet as of 2010 (Fig 3) [17]. Countries in blue or teal colours could reduce global land use by shifting to a USDA guideline diet (net positive land spared), while countries in green, red or yellow would cause an increase in land use (net negative land spared). Although 1 gigahectare of extra land would be required globally for a guideline diet (Fig 1), the world map shows how the results are much more variable at the country level. The countries that can spare the most land are the USA, Brazil and Australia. In contrast, the countries that require the most land to meet the guidelines are Mozambique, Saudi Arabia, and India. Global economic disparity is often described in terms of the gap between the Global North and the Global South. In contrast, our country-level map shows a strong hemispheric divide: the western hemisphere would largely spare global lands by shifting to a USDA guideline diet, whereas the eastern hemisphere would largely use up more global lands under such a diet. The Western hemisphere would spare significant amounts of land under a USDA diet largely due to current very high levels of meat consumption, via grain grown to feed livestock.

## Discussion

Currently, the world is in the midst of a “nutrition transition” that is marked by rapid changes in the composition and quantity of our diet [18]. In particular, around the world, diets are becoming more dominated by livestock, sugar, and saturated fat, and this is linked with the rising tide of obesity and diabetes [19]. These emerging diets are also linked with excessive land use and greenhouse gas emissions, the unsustainable use of water, and the loss of biodiversity [20]. Against this background of both unsustainable and unhealthy diets [21,22], nutritional guidelines such as those offered by the USDA show us what a balanced diet *ought* to look like.

Unfortunately, our analysis shows that there is not enough land for the world to adhere to the USDA guidelines under current agricultural practices. One gigahectare of fertile land—roughly the size of Canada—would be required. This is despite the fact that the USDA guideline diet is already less land-intensive than the current US diet. Our analysis also revealed a hemispheric divide. North America, South America and Oceania could spare significant amounts of land if they moved to the less meat-intensive (and consequently, grain-intensive) diet in the USDA guidelines. In contrast, Africa, the European Union and Asia would require a significant expansion of agricultural lands to support a USDA guideline diet. Further to this point, the fact that Europe is sparing land by avoiding a USDA guideline diet suggests that there may be sustainable ways to improve diets in the poorest countries avoid malnourishment, while also sparing land compared to the USDA guideline diet. Feeding the world while preserving natural land states and their ecosystem services is a complex problem that may require applying multiple solutions. We, therefore, conclude that revising national dietary guidelines to create dietary goals that are not just healthier but also more sustainable and equitable from a global land use perspective are part of the solution. In this way we build on the literature of the global land use community that discusses the challenge of maintaining ecosystem services while producing enough food to meet the global demand for nutrition [11]. The easy availability of FAO data helps makes this plausible.

However, it is worthwhile noting that reformulating national dietary guidelines with consideration of global land use also needs to account for cultural and economic variation in food sources. For instance, in the Global South, coarse grains (millet and sorghum), legumes, and game hunting are an important part of many diets [23]. However, these food sources are generally under-represented in datasets, suggesting more efforts should be targeted toward their data collection in order for land use estimation to become more accurate. This is an important area for future research. A full accounting of land use implications of dietary shifts including the full range of cultural and economic dietary heterogeneity is beyond the scope of our manuscript, since our more limited goal was only to establish why national-level dietary guidelines must go beyond nutritional health as a criterion to include land use as well, with the USDA guidelines representing an example of an unsustainable model.

The looming global land deficit suggested through this analysis is echoed by similar work on water [1,24]. Briefly, this literature points out that we also face the potential for widespread water shortages and that to avert such a crisis new paradigms are needed to conserve water and develop drought resistant crops and livestock. Another approach to reduce water use is through international trade to ensure that the food produced in regions where water is abundant can be traded with regions where water is scarce [25,26]. Sometimes this is called the trade in “virtual water” [27].

Our analysis was also broken down by continent and country. Recent dietary trends in Africa and Asia (Fig 2) show movement toward the UDSA guidelines, as reflected in other research on evolving diets in these regions [28]. China, India, and Saudi Arabia have changed most drastically in recent years with an increase in agricultural land use. Pakistan, along with India, is responding to growing consumer demand for more western diets by increasing beef production [29]. Of particular interest in Asia is China, which is rapidly increasing production in several sectors, largely contributing to Asia’s rapid agricultural growth rate (Fig A in S1 Appendix) [28]. Humans will have to deal with growing inequities as growing land use for meat consumption by rich countries causes rising food costs for staples such as pulses and grains and thus harms the poor and under-nourished remainder [30,31].

It is important to note that our analysis made simplifying assumptions and did not include all factors that could influence dietary and land use trends in coming years Our estimate is conservative since we relied upon recent historical data rather than attempting to project into the future using population models. The world’s population will continue increasing for years to come, creating stronger challenges than our analysis has described. On the other hand, by avoiding future projections, we also neglected new technologies and possible future increases in agricultural yield in continents like Africa.

The FAOSTAT dataset is a secondary data source and relies largely upon data collected from member countries. Therefore, it is subject to variable accuracy. This is reflected in our own analyses. For instance, at the global level, there should be no discrepancy between “total” and “domestic” land spared because total imports should, by definition, match total exports at the agglomerated global level. However, Fig 1A suggests a discrepancy between these two values of approximately 20% in 2010. This error could be due to a combination of factors, such as anomalous data points; differences between reported imports and exports (for instance, if a country under-reports imports or exports due to black market activity); or discrepancy between the FAO production data and the food balance sheets. We did not attempt country-level case studies to validate our results since it would be difficult to generalize from case studies to the overall accuracy of our findings. However, previous studies have compared results derived from FAOSTAT to remote sensing data [32] and IPCC data [33] for instance, finding fair but imperfect agreement between the data sources. Our finding that there is not enough land for the world to shift to a USDA guideline diet would likely not change if errors in the FAOSTAT dataset were removed. Therefore, our recommendation that national dietary guidelines should take global land use into consideration would likely also not change.

Our analysis concerns only masses and caloric values of food products, but a more detailed analysis would include more specific breakdowns of nutrients, fats and proteins. Similarly, differing demographics and their individual nutritional requirements were not accounted for. The FAO trade matrix could also be used in conjunction with country-specific yields to improve estimation of country-level land use, instead of using global average yields. These are valuable areas for future research.

Future research could also study the impact of real or potential dietary shifts on greenhouse gas emissions. Global agricultural production accounts for nearly 30% of total greenhouse gas (GHG) emissions [31]. Livestock alone are responsible for 18% of GHG emissions, which is higher than the share of GHG emissions from transportation [29]. Hence, a shift to less meat consumption would also reduce GHG emissions. Other topics for future research include the effects of food lost during storage and transportation and (more importantly) food lost through waste and disposal. Food loss is significant around the world, thus reducing food loss could also help spare land.

The implication of our results is that countries should coordinate their formulation of dietary guidelines such that they are based not only on health considerations but also consideration of sustainable global land use, equity, and natural ecosystem conservation. Moreover, given that international agricultural trade is growing and global lands are increasingly in demand for growing food, international coordination should incentivize country-level improvements in dietary habits that result in global land sparing, similar to how countries are beginning to coordinate reductions in their greenhouse gas emissions.

## Supporting information

S1 Appendix Text A and B, Tables A and B, Fig A. (PDF)
