# Correction: Global land use implications of dietary trends

**DOI:** 10.1371/journal.pone.0205312

**Published:** 2018-10-03

**Authors:** Sarah Rizvi, Chris Pagnutti, Evan Fraser, Chris T. Bauch, Madhur Anand

In the Author Contributions section, Chris Pagnutti (CP) should be listed as one of the persons who wrote the original draft of the paper.
