# Supplementary material for: Global land use implications of dietary trends
Source: PLoS One. 2018 Aug 8;13(8):e0200781. doi: 10.1371/journal.pone.0200781 (PMC6082513; doi:10.1371/journal.pone.0200781)

Global Land Use Implications of Dietary Shifts:

Sarah Rizvi, Chris Pagnutti, Evan Fraser, Chris T. Bauch, Madhur Anand

Supporting Information: S1 Appendix

Table of Contents

**Item**	**Page**
Text A: Methods used to compute livestock yield (Text S1)	1
Text B: Python Code for analysing land use change under USDA guidelines (Text S2)	7
Table A: FAO items, codes and the corresponding food group	21
Table B: Top livestock product producing countries on average since 1990	21
Fig A: Analysis by continent	22

Text A: Methods used to compute livestock yield

We aim to extend the traditional definition of crop yield to apply to livestock products. In the context of crops, if p is the quantity produced, and a is the area of land used to produce the quantity p, then yield y is defined as

$$y = p/a$$

In the remainder of this section we use the following labels: i denotes an animal product (e.g. cattle meat, milk, etc.), j denotes an animal meat product (e.g. cattle meat), k denotes a non-meat animal product (i.e. milk, eggs), and l denotes a crop (e.g. maize, oats, etc.).

Production

The FAO reports annual data for country, sub-continent, continent, and global-level production of various livestock products. Let us denote this quantity by p_i where i labels a given animal product (e.g. cattle meat).

Adjusting production for import and export of live animals

At any scale smaller than the global one (e.g. country-level), the concept of production can be defined in several ways. For example, we may define production simply as p_i; that is, the quantity reported to have been produced in a given country. But consider production of cattle meat for a moment. In some cases it may be useful to regard exports of live cattle as contributing to a country's actual beef production. After all, the country used land to produce the cattle that was exported and so should be considered in the calculation of yield. If we let $N^{(E)}_j$ denote the number of live animals exported of the type that produce a meat product j, and we let m_j denote the average carcass weight, then we can define an adjusted meat production $p^{(E)}_j$ to be

$p^{(E)}{}_j = p_j + N^{(E)}{}_j m_j$

On the other hand, suppose a given country imports a number $N^{(I)}{}_j$ of animals that are slaughtered for meat. Then we should subtract this meat from the country's overall production because they did not use their own land to produce it. That is, we define another adjusted production $p^{(IE)}{}_j$ to be

$p^{(IE)}{}_j = p_j + (N^{(E)}{}_j - N^{(I)}{}_j)m_j$

This definition assumes that all imported animals are slaughtered for meat within the year in which the trade is reported.

Adjusting production for culling of dairy/egg animals

Finally, there are cases when we would like to exclude meat produced from the culling of dairy or egg animals. If we denote culling rate by r_k of dairy or egg animals labeled by k (e.g. dairy cattle) but of the same type that produce a meat product j (e.g. meat cattle), then the adjusted production $p^{(IEC)}{}_j$:

$p^{(IEC)}{}_i = p_i + (N^{(E)}{}_i - N^{(I)}{}_i - r_k N_k)m$

where N_k is the number of producing dairy or egg animals in a given year. This definition of production is useful when determining the amount of feed required to produce a given quantity of meat since the feed given to these culled animals was primarily used for milk or egg production.

The methods we used to estimate r_k is differ for dairy and egg animals. Let us define the following quantities: $N^{(t)}$ is the stock of live animals (e.g. cattle) reported by a given country for the year t; $I^{(t)}$ and $E^{(t)}$ are the number of imported and exported live animals respectively; $D^{(t)}=N^{(t)} + I^{(t)} - E^{(t)}$ is the number of domestic live animals; $S^{(t)}$ is the number of animals slaughtered for meat; $P^{(t)}$ is the number of animals producing milk or eggs.

Culling rate for dairy animals

The lifespan of typical dairy animals (e.g. cows) is greater than one year, which is the time resolution of the FAO data. For cattle, the number of animals not slaughtered is $D^{(t)}$ - $S^{(t)}$. Thus, we estimate the number of cattle derived from cattle births from the previous year is

$B^{(t)} = N^{(t)} - (D^{(t-1)} - S^{(t-1)})$

The number of animals born into the dairy sector is assumed to be in the same proportion as the number of dairy animals relative to the domestic animal population. That is, the number of new dairy animals is given by

$B_P{}^{(t)} = B^{(t)}P^{(t)}/D^{(t)}$

Thus, the number of dairy animals in a given year that were present in the previous year is given by

$P_O{}^{(t)} = P^{(t)} - B_P{}^{(t)}$

Finally, this implies that the number of dairy animals culled in a given year is given by

$C^{(t)} = P^{(t)} - P_O^{(t+1)}$

so the culling rate is

$r_k^{(t)} = C^{(t)}/P^{(t)}$

To check the validity of this method we can use the United States as an example. Our method gives average cull rate of 0.35 for U.S. dairy cattle, which is in excellent agreement with the value of 0.36 reported by the USDA[1]. Also, as the slaughter of cattle is forbidden in many states in India, we should expect a low culling rate there[2]. We find an average culling rate for dairy cattle in India of about 0.04.

Culling rate for egg-laying animals

The method to estimate the culling rate of egg-laying animals (e.g. hens) differs from that for dairy animals because, although the lifespan of egg-laying animals is typically longer than one year, that of a meat bird is typically much less than a year. In this case, we define the cumulative stock to be the sum of the number of animals slaughtered and the following year's stock:

$A^{(t)} = S^{(t)} + N^{(t+1)}$

Then the number of new births in a given year is

$B^{(t)} = A^{(t)} - D^{(t)}$

and number of animals born into the egg sector is

$B_P^{(t)} = B^{(t)}P^{(t)}/A^{(t)}$

Then the number of egg-laying animals in a given year that remain from the previous year is

$P_O^{(t)} = P^{(t)} - B_P^{(t-1)}$

Finally, this implies that the number of egg-laying animals culled in a given year is given by

$C^{(t)} = P^{(t)} - P_O^{(t+1)}$

so the cull rate is

$r_c^{(t)} = C^{(t)}/P^{(t)}$

Again, we can check the method's results against what might be expected from known practices in the United States. There, and in much of the developed world, egg-laying hens are typically slaughtered at less than two years of age[3]. Thus, if we assume uniform age distribution, we expect a culling rate greater than 0.5 but less than one. For the U.S. we find an average culling rate for egg-laying hens of about 0.8. Moreover, we find a smaller culling rate in developing countries, where replacement hens may be less available and/or not economically viable.

Dividing production into pastoral and mixed/landless production

For ruminants, we used estimates of the quantity produced in both the pastoral and mixed/landless agricultural systems at the sub-continent level[4]. Using a quadratic interpolant on the relative production in these two systems, we divide the production p_i of ruminant animal products into two parts: production in the pastoral system $p^{(P)}_i$, $p^{(P,E)}_i$, $p^{(P,IE)}_i$, $p^{(P,IEC)}_i$, and production in the mixed/landless system $p^{(ML)}_i$, $p^{(ML,E)}_i$, $p^{(ML,IE)}_i$, $p^{(ML,IEC)}_i$.

In the event that production data is missing from the FAOSTAT for a given country and year, we estimate the production to be the same as that in either the next or previous year in which production data was reported. If no such data can be found, we assume that the production is zero.

Land area used for meat production

The land used to produce animal products is divided into two parts: pasture area and cropland used to produce feed.

Ruminant production and pasture area

Ruminant livestock such as cattle and sheep require pasture for their production. Data for the total pasture area A_P used for agricultural production is provided in the FAOSTAT database at the country, sub-continent, continent and global levels. If no such data can be found, we assume that the pasture area is 69% (world average) of the country's agricultural area. To determine the pasture area required to produce a given product (e.g. cattle meat) in a given country, we first divided the pasture area into two parts: pasture area in the pastoral system, and pasture area in the mixed/landless system, denoted by $A^{(P)}$ and $A^{(ML)}$ respectively. This was done using a quadratic interpolant on estimates of grass land areas in these two agricultural systems in the corresponding sub-continent[4].

Next, let us denote the stocks of animal i in livestock units by U_i where i labels one of the following animals: meat cattle, dairy cattle, meat buffalo, dairy buffalo, horses, asses, mules, meat sheep, dairy sheep, meat goats, meat camels, dairy camels, and camelids[5]. The stocks of meat animals is defined to be the total number of animals minus the number of dairy or egg animals, where applicable. We further divided those stocks into parts found in the pastoral and mixed/landless systems, denoted by $U^{(P)}_i$ and $U^{(\mathrm{ML})}_i$ respectively. The latter was again done using a quadratic interpolant on estimates of the proportions of each livestock population that is in either production system in the corresponding sub-continent. The proportions were inferred from the production, carcass weight/production per animal, and off-take rates in each production system[4]. Then the area of pasture in the mixed/landless system assigned to an animal n is the total area of pasture in the mixed/landless system times the fraction of all livestock units represented by meat cattle. That is, the pasture area in the pastoral and mixed/landless system assigned to the production of an animal product i is given by

$$A^{(ML)}_i = A^{(ML)} U^{(ML)}_i / \Sigma_i U^{(ML)}_i$$

and

$$A^{(P)}_i = A^{(P)} U^{(P)}_i / \Sigma_i U^{(P)}_i$$

respectively.

Cropland area used for feed

Ruminant livestock in the mixed/landless system as well as non-ruminant livestock, including pigs and chickens, consume feed. This feed can be composed of grasses (for ruminants), crop residues and food crops. The land required to produce the grasses used in ruminant feed is already accounted for in the pasture area. We regard the land used to produce the residue portion of feed to be zero since that land was primarily used for a different purpose (e.g. crop production). Thus, here we are interested in the area of cropland used to produce the proportion of feed represented by food crops.

Bouwman et al. give estimates of the feed conversion rate $r^{(f)}_i$ at the sub-continent level for a given animal product labeled by i; that is, the number of units of feed (dry matter) required to produce one unit of the animal product[4]. They also give estimates of the proportion of feed f_i represented by food crops for a given animal product. Using a quadratic interpolant on these data we can estimate the quantity of food crops required to produce a unit of the animal product by $p^{(ML,IEC)}_{ij} f_i r^{(f)}_i$. However, we do not use this value, which for the moment we will call the un-normalized feed quantity, for the feed quantity because we do not have a reliable way to convert dry matter to harvest weight, the latter of which is reported in the FAOSTAT database, and we could not find any data on the time dependence of feed composition[6]. Instead, for a given animal product i, we estimated the proportion p_i of the total available feed quantity that is assigned to the production of that product to be the proportion of the total feed quantity represented by the animal product i. That is,

$$p_i = p^{(ML,IEC)}_i f_i r^{(f)}_i \, / \, \Sigma_i \, p^{(ML,IEC)}_i f_i r^{(f)}_i$$

For any given crop the quantity of that crop in a country's supply that is available for livestock feed is reported in the commodity balances of the FAOSTAT database. Let Q_j denote the quantity of crop labeled by j assigned to feed. Data for the country's production P_j, imports I_j and exports E_j of a crop j are given in the FAOSTAT database, which can be used to determine the country's self-sufficiency ratio s_j for that crop,

$$s_j = P_j \, /(P_j + I_j - E_j)$$

Then the quantity of that feed q_{ij} that is assigned to the production of a given animal product i is given by

$$q_{ij} = p_i \, s_j \, Q_j$$

The factor s_j accounts for the fact that land is not required to produce feed that imported by a given country. Finally, the yield y_j of crop j is given in the FAOSTAT database, so the area of cropland required to produce the amount of that crop that was produced domestically for feed that was used to produce the animal product i is given by

$$A^{(C)}_{ij} = q_{ij} \, / \, y_j$$

In the event that there is insufficient data in the FAOSTAT database to complete the calculation for a given country and year, we estimate the area to be the same as that in either the next or previous year in which sufficient data was reported.

Yield

With the above definitions, calculating the yield y_i of an animal product in the sense of production per hectare of land used is straightforward.

$$y_i = (p^{(P,IE)}{}_i + p^{(ML,IE)}{}_i)/ (\Sigma_j A^{(C)}{}_{ij} + A^{(P)}{}_i + A^{(ML)}{}_i)$$

We also define yield with respect to cropland use only by

$$y^{(C)}{}_i = p^{(ML,IE)}{}_i / \Sigma_j A^{(C)}{}_{ij}$$

In the event that there is insufficient data to complete the calculation in the FAOSTAT data for a given country and year, we estimate the yield to be the same as that in either the next or previous year in which sufficient data was reported. If no such data can be found, we assume that the yield is the same as that of the sub-continent to which the country belongs.

References

1 United States Department of Agriculture (2013) "Livestock Slaughter" and "Poultry Slaughter" and ERS calculations for commercial slaughter by class. Available at http://www.ers.usda.gov/datafiles/Livestock_Meat_Domestic_Data/Meat_statistics/Livestock_and_poultry_slaughter/SlaughterCounts.xls

2 Rahman A (2007) (ed Global Campaign Coalition Against the Long Distance Transport of Animals for Slaughter - Asia Status Report).

3 Beutler A (2007) Introduction to poultry production in Saskatchewan (Ministry of Agriculture. Government of Saskatchewan). Available at http://www.agriculture.gov.sk.ca/introduction_poultry_production_saskatchewan.

4 Bouwman AF, Van der Hoek KW, Eickhout B, Soenario I (2005) Exploring changes in world ruminant production systems. *Agricultural Systems* 84:121-153.

5 Food and Agriculture Organization of the United Nations (2003) Compendium of Agricultural – Environmental Indicators (ed FAO Statistics Division, Rome)

6 Wirsenius S (2000) Human use of land and organic materials: modeling the turnover of biomass in the global food system (Chalmers University of Technology, Gothenburg, Sweden).

<u>Text B:</u> Python code used for computing the change in land use under the assumption that a given country/region were to follow the USDA guidelines.

Here we present the Python code used for computing the change in land use under the assumption that a given country/region were to follow the USDA guidelines. The following code depends on the FAOTools library available at https://github.com/Pacopag/faolyzer, and is compatible with Python2.7.

```
import sys
sys.path.append('../faotools/')
from FAOTools import *

calorie_intakes = {
        1000 : {
                'fruits':93.12,
                'vegetables':34.18,
                'grains':266,
                'meats':108.67,
                'dairy':484.67,
                'oils':199,
                'sugar':165},

        2000 : {
                'fruits':186.24,
                'vegetables':87,
                'grains':532,
                'meats':298.84,
                'dairy':727,
                'oils':239,
                'sugar':267}
}
cereal_codes_balance = [2905]
fruit_codes_balance = [2919]
oil_codes_balance = [2913]
meat_codes_balance = [2911]
vegetable_codes_balance = [2907,2918]
sugar_codes_balance = [2909]
butter_code = 2740
milk_code = 2948

food_groups = {
```

```
        'fruits'  :[2919,2655],
        'vegetables':[2918,2907],
        'grains' :[2905,2656,2658],
        'meats'         :[2911,2912,2731,2732,2733,2734,2913,2949],
        'dairy'         :[2948, 2740],
        'oils'          :[2914],
        'sugar'         :[2908,2909,2922]
}


b2p_mappings = {
        2656:[44],              #beer
        2658:[1717],            #alcohol
        2655:[560],             #wine
        2905:[1717],            #cereal
        2919:[1801],            #fruit
        2913:[1732],            #oilcrops
        2911:[1726],            #pulses
        2907:[1720],            #roots
        2909:[156,157,161],     #sugar
        2908:[156,157,161],     #sugarcrops
        2912:[1729],            #treenuts
        2918:[1735],            #vegetables
        2922:[661,656],                 #stimulants
        2914:[1732],            #oils
        2731:[867],             #beef
        2732:[977,1017],        #mutton
        2733:[1035],            #pork
        2734:[1058],            #poultry
        2948:[882],             #milk
        2740:[886],             #butter
        2949:[1062],            #eggs
}
b2p_conversions = {
        2656:4.78,      #beer
        2658:0.6,       #alcohol
        2655:0.7,       #wine
        2905:1.0,       #cereal
        2919:1.0,       #fruit
        2913:1.0,       #oilcrops
        2911:1.0,       #pulses
```

```
        2907:1.0,       #roots
        2909:0.12,      #sugar
        2908:1.0,       #sugarcrops
        2912:1.0,       #treenuts
        2918:1.0,       #vegetables
        2922:1.0,       #stimulants
        2914:0.2,       #oils
        2731:1.0,       #beef
        2732:1.0,       #mutton
        2733:1.0,       #pork
        2734:1.0,       #poultry
        2948:1.0,       #milk
        2740:0.047,     #butter
        2949:1.0,       #eggs
}
foods_balanced = b2p_mappings.keys()
fbs_codes = [food_code_fb,food_supply_code_fb,domestic_supply_code_fb,import_code_fb]
#parts of food balance sheet that interest us.

results_groups = {'fruits':[],'vegetables':[],'grains':[],'meats':[],'dairy':[],'oils':[],'sugar':[]}
results_groups_imports =
{'fruits':[],'vegetables':[],'grains':[],'meats':[],'dairy':[],'oils':[],'sugar':[]}
results_groups_domestic =
{'fruits':[],'vegetables':[],'grains':[],'meats':[],'dairy':[],'oils':[],'sugar':[]}
results_total = []
results_total_imports = []
results_total_domestic = []

def get_land_saved_by_food_guide(year,country_code,calorie_level=2000):

        start_year = 1961
        end_year = 2009
        if country_code < world_code:
                spec = {'countrycode':country_code}
                fields = {'start_year':1,'end_year':1}
                rec,f = find_one(table_balancers,spec,fields)
                if rec is None:
                        print "Country not found",country_code
                        raise ValueError
                start_year = 1961 if rec['start_year']=='' else rec['start_year']
                end_year = 2009 if rec['end_year']=='' else rec['end_year']
```

```
	if year<start_year:
		return get_land_saved_by_food_guide(start_year,country_code)
	elif year>end_year:
		return get_land_saved_by_food_guide(end_year,country_code)

	tot_land_saved = 0.0
	tot_land_saved_imports = 0.0
	tot_land_saved_domestic = 0.0

	#Pre-fetch world-average yields to use as estimated land use due to imports.
	world_yields = {}
	for fb in foods_balanced:
		pcs = b2p_mappings[fb] if fb!=butter_code else b2p_mappings[milk_code]
		yld,f = get_weighted_yield(year,world_code,pcs)
		world_yields[fb] = yld
		#print "World yield ",yld

	spec = {'year':year, 'countrycode':country_code, 'itemcode':population_item_code,
'elementcode':population_element_code}
	fields = {'value':1}
	rec,f = find_one(table_population, spec, fields, [],'year', None)
	pop = 1000.0*rec['value'] #Population is reported in units of 1000 people, hence the
conversion.

	#print "Population\t".pop."\n"
	for fg in food_groups:
		res_land_saved = 0.0
		res_land_saved_imports = 0.0
		res_land_saved_domestic = 0.0

		# Get country's "weighting" trend for the group components (e.g. %wine and
%fruit for "fruits" group)
		group_components = food_groups[fg]
		component_weights = {}
		component_total = 0.0
		spec = {'year':year, 'countrycode':country_code,
'itemcode':{'$in':group_components}, 'elementcode':food_supply_code_fb}
		fields = {'itemcode':1,'value':1}
		qry,f = find(table_foodbalance,spec,fields)
		for r in qry:
```

```
				v = r['value'] if bool(r['value']) else 0.0
				component_weights[r['itemcode']] = v
				component_total += v

			for fb in group_components: #(foods_balanced as fb) {
				#print "------------------------------------------\n"
				component_weights[fb] = component_weights[fb]/component_total if fb
in component_weights and component_total!=0 else 0.0  #Normalizes the component weights.
				#print "Component weight ".component_weights[fb]."\n"
				spec = {'year':year, 'countrycode':country_code, 'itemcode':fb,
'elementcode':{'$in':fbs_codes}}
				fields = {'elementcode':1,'value':1}
				qry,f = find(table_foodbalance, spec, fields)
				(food,domestic,imports,supply) = (0.0,0.0,0.0,0.0)

				for r in qry:
					v = float(r['value']) if r['value']!='' else 0.0
					if r['elementcode']==food_code_fb:
					#Note: food balance sheets report quantities in kilotonnes
					#	so we multiply by 1000.0 to convert to tonnes.
						food = 1000.0*v
					elif r['elementcode']==domestic_supply_code_fb:
						domestic = 1000.0*v
					elif r['elementcode']==import_code_fb:
						imports = 1000.0*v
					elif r['elementcode']==food_supply_code_fb:
						supply = v

				#print "Food\t\t".food."\n"
				#print "Supply\t\t".supply."\n"
				#print "Domestic\t".domestic."\n"
				#print "Import\t\t".import."\n"

				idr = imports/domestic if domestic!=0 else 1.0
				import_adj = (idr*food)/b2p_conversions[fb]
				food_adj = (food - import_adj)/b2p_conversions[fb]

				#print "IDR\t".idr."\n"
				#print "Food (adj)\t".food_adj."\n"
				#print "Import (adj)\t".import_adj."\n"
```

```
			pcs = b2p_mappings[fb] if fb!=butter_code else
b2p_mappings[milk_code]
			yld,f = get_weighted_yield(year,country_code,pcs)
			if not bool(yld):
				idr = 1.0
				import_adj = import_adj + food_adj
				food_adj = 0.0
				yld = 1.0#just a dummy value
			#print "Yield ".country_code.":".implode(",",pcs).":".yield."\n"
			land_local = food_adj/yld
			#print "Local land use ".land_local."\n"
			if world_yields[fb]==0:
				print year,fb
			land_remote = import_adj/world_yields[fb]
			#print "Remote land use ".land_remote."\n"
			land_total = land_local+land_remote
			#print "Total land use ".land_total."\n"
			rec_cal = component_weights[fb]*calorie_intakes[calorie_level][fg]
			#print "Recommended daily calories ".rec_cal."\n"
			#print "Population ".pop."\n"
			tot_rec_cal = rec_cal*pop*365
			#print "Recommended annual calories ".tot_rec_cal."\n"
			kg_per_cal = food/(supply*pop*365) if tot_rec_cal!=0 else 0.0
			#print "kg per cal ".kg_per_cal."\n"
			tot_rec_kg = tot_rec_cal*kg_per_cal
			#print "Recommended kg food ".tot_rec_kg."\n"
			rec_import = idr*tot_rec_kg
			#print "Recommended import ".rec_import."\n"
			rec_food = tot_rec_kg - rec_import
			#print "Recommended food (local)".rec_food."\n"
			rec_land_local = rec_food/yld if bool(yld) else 0.0
			#print "Recommended local land use ".rec_land_local."\n"
			rec_land_remote = rec_import/world_yields[fb]
			#print "Recommended remote land use ".rec_land_remote."\n"
			rec_land_total = rec_land_local+rec_land_remote
			#print "Recommended total land use ".rec_land_total."\n"
			diff_land_local = land_local-rec_land_local
			#print "Local land saved ".diff_land_local."\n"
			diff_land_remote = land_remote-rec_land_remote
			#print "Remote land saved ".diff_land_remote."\n"
			diff_land_total = land_total-rec_land_total
```

```
                        #print "Total land saved ".diff_land_total."\n"

                        res_land_saved += diff_land_total
                        res_land_saved_imports += diff_land_remote
                        res_land_saved_domestic += diff_land_local
                        tot_land_saved += diff_land_total
                        tot_land_saved_imports += diff_land_remote
                        tot_land_saved_domestic += diff_land_local

                results_groups[fg] = res_land_saved
                results_groups_imports[fg] = res_land_saved_imports
                results_groups_domestic[fg] = res_land_saved_domestic
        # end foreach food_groups
        results_total = tot_land_saved
        results_total_imports = tot_land_saved_imports
        results_total_domestic = tot_land_saved_domestic


        return {

        'total':{'total':results_total,'local':results_total_domestic,'remote':results_total_imports}
,

        'fruits':{'total':results_groups['fruits'],'local':results_groups_domestic['fruits'],'remote':r
esults_groups_imports['fruits']},

        'vegetables':{'total':results_groups['vegetables'],'local':results_groups_domestic['vegeta
bles'],'remote':results_groups_imports['vegetables']},

        'grains':{'total':results_groups['grains'],'local':results_groups_domestic['grains'],'remote'
:results_groups_imports['grains']},

        'oils':{'total':results_groups['oils'],'local':results_groups_domestic['oils'],'remote':results
_groups_imports['oils']},

        'discretional':{'total':results_groups['sugar'],'local':results_groups_domestic['sugar'],'re
mote':results_groups_imports['sugar']},

        'meats':{'total':results_groups['meats'],'local':results_groups_domestic['meats'],'remote
':results_groups_imports['meats']},
```

'dairy':{'total':results_groups['dairy'],'local':results_groups_domestic['dairy'],'remote':results_groups_imports['dairy']},
}

As an example, suppose we wish to know the change in land use if the United States (FAO country code 231) were to follow the guidelines in the year 2003. From a Python2.7 interpreter, we could run the following commands:

```
>>> import foodguide
>>> foodguide.get_land_saved_by_food_guide(2003,  231)
```

The output is a dictionary indexed by each food group. For example,

{'oils': {'remote': 11935955.053925896, 'total': 17516498.66811648, 'local': 5580543.614190584}, 'dairy': {'remote': -894147.8366835385, 'total': -16054304.588332873, 'local': -15160156.751649339}, 'discretional': {'remote': 2765292.0805478753, 'total': 2800929.6844484867, 'local': 35637.6039006114}, 'grains': {'remote': -72896.05288693658, 'total': 378229.8886981746, 'local': 451125.94158511085}, 'fruits': {'remote': -593218.3968910384, 'total': -941216.3453534044, 'local': -347997.94846236636}, 'vegetables': {'remote': 288021.3276913505, 'total': 1127656.2250690062, 'local': 839634.8973776556}, 'total': {'remote': 33799978.598322116, 'total': 128289100.90765314, 'local': 94489122.309331}, 'meats': {'remote': 20370972.422618505, 'total': 123461307.37500726, 'local': 103090334.95238875}}

Figure Legends

```
cereal_codes_balance = [2905]
fruit_codes_balance = [2919]
oil_codes_balance = [2913]
meat_codes_balance = [2911]
vegetable_codes_balance = [2907,2918]
sugar_codes_balance = [2909]

butter_code = 2740
milk_code = 2948

food_groups = {

 'fruits'  :[2919,2655],
```

```
 'vegetables':[2918,2907],
 'grains'  :[2905,2656,2658],
 'meats'    :[2911,2912,2731,2732,2733,2734,2913,2949],
 'dairy'    :[2948, 2740],
 'oils'    :[2914],
 'sugar'    :[2908,2909,2922]

}


b2p_mappings = {
 2656:[44],  #beer       *
 2658:[1717],  #alcohol    *
 2655:[560],  #wine      **
 2905:[1717],  #cereal      *
 2919:[1801],  #fruit       **
 2913:[1732],  #oilcrops    ***
 2911:[1726],  #pulses    *** ***
 2907:[1720],  #roots      ****
 2909:[156,157,161],  #sugar      *****
 2908:[156,157,161],  #sugarcrops      *****
 2912:[1729],  #treenuts    *** ***
 2918:[1735],  #vegetables  ****
 2922:[661,656],  #stimulants  *****
 2914:[1732],  #oils        ***
 2731:[867],  #beef       *** ***
 2732:[977,1017],  #mutton  *** ***
 2733:[1035],  #pork      *** ***
 2734:[1058],  #poultry    *** ***
 2948:[882],    #milk      *** *** *
 2740:[886],    #butter    *** *** *
 2949:[1062],    #eggs    *** ***


}

b2p_conversions = {
 2656:4.78,  #beer
 2658:0.6,  #alcohol
 2655:0.7,  #wine
 2905:1.0,  #cereal
 2919:1.0,  #fruit
 2913:1.0,  #oilcrops
 2911:1.0,  #pulses
 2907:1.0,  #roots
 2909:0.12,  #sugar
```

```
 2908:1.0,  #sugarcrops
 2912:1.0,  #treenuts
 2918:1.0,  #vegetables
 2922:1.0,  #stimulants
 2914:0.2,  #oils
 2731:1.0,  #beef
 2732:1.0,  #mutton
 2733:1.0,  #pork
 2734:1.0,  #poultry
 2948:1.0,  #milk
 2740:0.047,  #butter
 2949:1.0,  #eggs
}


calorie_intakes = {
 1000 : {
   'fruits':93.12,
   'vegetables':34.18,
   'grains':266,
   'meats':108.67,
   'dairy':484.67,
   'oils':199,
   'sugar':165},

 2000 : {
   'fruits':186.24,
   'vegetables':87,
   'grains':532,
   'meats':298.84,
   'dairy':727,
   'oils':239,
   'sugar':267}
}
import sys
sys.path.append('../faotools/')
from FAOTools import *
from calorie_intakes import *
from code_defns import *

foods_balanced = b2p_mappings.keys()
fbs_codes = [food_code_fb,food_supply_code_fb,domestic_supply_code_fb,import_code_fb]
#parts of food balance sheet that interest us.

results_groups = {'fruits':[],'vegetables':[],'grains':[],'meats':[],'dairy':[],'oils':[],'sugar':[]}
results_groups_imports = {'fruits':[],'vegetables':[],'grains':[],'meats':[],'dairy':[],'oils':[],'sugar':[]}
```

```
results_groups_domestic =
{'fruits':[],'vegetables':[],'grains':[],'meats':[],'dairy':[],'oils':[],'sugar':[]}
results_total = []
results_total_imports = []
results_total_domestic = []

def get_land_saved_by_food_guide(year,country_code,calorie_level=2000):

 start_year = 1961
 end_year = 2009
 if country_code < world_code:
   spec = {'countrycode':country_code}
   fields = {'start_year':1,'end_year':1}
   rec,f = find_one(table_balancers,spec,fields)
   if rec is None:
     print "Country not found",country_code
     raise ValueError
   start_year = 1961 if rec['start_year']=='' else rec['start_year']
   end_year = 2009 if rec['end_year']=='' else rec['end_year']

 if year<start_year:
   return get_land_saved_by_food_guide(start_year,country_code)
 elif year>end_year:
   return get_land_saved_by_food_guide(end_year,country_code)

 tot_land_saved = 0.0
 tot_land_saved_imports = 0.0
 tot_land_saved_domestic = 0.0

 #Pre-fetch world-average yields to use as estimated land use due to imports.
 world_yields = {}
 for fb in foods_balanced:
   pcs = b2p_mappings[fb] if fb!=butter_code else b2p_mappings[milk_code]
   yld,f = get_weighted_yield(year,world_code,pcs)
   world_yields[fb] = yld
   #print "World yield ",yld

 spec = {'year':year, 'countrycode':country_code, 'itemcode':population_item_code,
'elementcode':population_element_code}
 fields = {'value':1}
 rec,f = find_one(table_population, spec, fields, [],'year', None)
 pop = 1000.0*rec['value'] #Population is reported in units of 1000 people, hence the conversion.

 #print "Population\t".pop."\n"
 for fg in food_groups:
   res_land_saved = 0.0
```

```
  res_land_saved_imports = 0.0
  res_land_saved_domestic = 0.0

  # Get country's "weighting" trend for the group components (e.g. %wine and %fruit for "fruits"
group)
  group_components = food_groups[fg]
  component_weights = {}
  component_total = 0.0
  spec = {'year':year, 'countrycode':country_code, 'itemcode':{'$in':group_components},
'elementcode':food_supply_code_fb}
  fields = {'itemcode':1,'value':1}
  qry,f = find(table_foodbalance,spec,fields)
  for r in qry:
    v = r['value'] if bool(r['value']) else 0.0
    component_weights[r['itemcode']] = v
    component_total += v

  for fb in group_components: #(foods_balanced as fb) {
    #print "------------------------------------------\n"
    component_weights[fb] = component_weights[fb]/component_total if fb in
component_weights and component_total!=0 else 0.0 #Normalizes the component weights.
    #print "Component weight ".component_weights[fb]."\n"
    spec = {'year':year, 'countrycode':country_code, 'itemcode':fb,
'elementcode':{'$in':fbs_codes}}
    fields = {'elementcode':1,'value':1}
    qry,f = find(table_foodbalance, spec, fields)
    (food,domestic,imports,supply) = (0.0,0.0,0.0,0.0)
    for r in qry:
      v = float(r['value']) if r['value']!='' else 0.0
      if r['elementcode']==food_code_fb:
      #Note: food balance sheets report quantities in kilotonnes

#      so we multiply by 1000.0 to convert to tonnes.
        food = 1000.0*v
      elif r['elementcode']==domestic_supply_code_fb:
        domestic = 1000.0*v
      elif r['elementcode']==import_code_fb:
        imports = 1000.0*v
      elif r['elementcode']==food_supply_code_fb:
        supply = v

    #print "Food\t\t".food."\n"
    #print "Supply\t\t".supply."\n"
    #print "Domestic\t".domestic."\n"
    #print "Import\t\t".import."\n"
```

```
    idr = imports/domestic if domestic!=0 else 1.0
    import_adj = (idr*food)/b2p_conversions[fb] #import_adj now means imported "food".
    food_adj = (food - import_adj)/b2p_conversions[fb] #food_adj now means locally produced
"food".

    #print "IDR\t".idr."\n"
    #print "Food (adj)\t".food_adj."\n"
    #print "Import (adj)\t".import_adj."\n"

    pcs = b2p_mappings[fb] if fb!=butter_code else b2p_mappings[milk_code]
    yld,f = get_weighted_yield(year,country_code,pcs)
    if not bool(yld):
      idr = 1.0 # Production comes from processed imports (e.g. Canada and Sugars)
      import_adj = import_adj + food_adj
      food_adj = 0.0
      yld = 1.0#just a dummy value
    #print "Yield ".country_code.":".implode(",",pcs).":".yield."\n"
    land_local = food_adj/yld
    #print "Local land use ".land_local."\n"
    if world_yields[fb]==0:
      print year,fb
    land_remote = import_adj/world_yields[fb]
    #print "Remote land use ".land_remote."\n"
    land_total = land_local+land_remote
    #print "Total land use ".land_total."\n"
    rec_cal = component_weights[fb]*calorie_intakes[calorie_level][fg]
    #print "Recommended daily calories ".rec_cal."\n"
    #print "Population ".pop."\n"
    tot_rec_cal = rec_cal*pop*365
    #print "Recommended annual calories ".tot_rec_cal."\n"
    kg_per_cal = food/(supply*pop*365) if tot_rec_cal!=0 else 0.0
    #print "kg per cal ".kg_per_cal."\n"
    tot_rec_kg = tot_rec_cal*kg_per_cal
    #print "Recommended kg food ".tot_rec_kg."\n"
    rec_import = idr*tot_rec_kg
    #print "Recommended import ".rec_import."\n"
    rec_food = tot_rec_kg - rec_import
    #print "Recommended food (local)".rec_food."\n"
    rec_land_local = rec_food/yld if bool(yld) else 0.0
    #print "Recommended local land use ".rec_land_local."\n"
    rec_land_remote = rec_import/world_yields[fb]
    #print "Recommended remote land use ".rec_land_remote."\n"
    rec_land_total = rec_land_local+rec_land_remote
    #print "Recommended total land use ".rec_land_total."\n"
    diff_land_local = land_local-rec_land_local
    #print "Local land saved ".diff_land_local."\n"
```

```
    diff_land_remote = land_remote-rec_land_remote
    #print "Remote land saved ".diff_land_remote."\n"
    diff_land_total = land_total-rec_land_total
    #print "Total land saved ".diff_land_total."\n"

    res_land_saved += diff_land_total
    res_land_saved_imports += diff_land_remote
    res_land_saved_domestic += diff_land_local
    tot_land_saved += diff_land_total
    tot_land_saved_imports += diff_land_remote
    tot_land_saved_domestic += diff_land_local

  results_groups[fg] = res_land_saved
  results_groups_imports[fg] = res_land_saved_imports
  results_groups_domestic[fg] = res_land_saved_domestic
 # end foreach food_groups
 results_total = tot_land_saved
 results_total_imports = tot_land_saved_imports
 results_total_domestic = tot_land_saved_domestic

 return {
    'total':{'total':results_total,'local':results_total_domestic,'remote':results_total_imports},
    'fruits':{'total':results_groups['fruits'],'local':results_groups_domestic['fruits'],'remote':results_
groups_imports['fruits']},
    'vegetables':{'total':results_groups['vegetables'],'local':results_groups_domestic['vegetables'],'r
emote':results_groups_imports['vegetables']},
    'grains':{'total':results_groups['grains'],'local':results_groups_domestic['grains'],'remote':result
s_groups_imports['grains']},
    'oils':{'total':results_groups['oils'],'local':results_groups_domestic['oils'],'remote':results_group
s_imports['oils']},
    'discretional':{'total':results_groups['sugar'],'local':results_groups_domestic['sugar'],'remote':re
sults_groups_imports['sugar']},
    'meats':{'total':results_groups['meats'],'local':results_groups_domestic['meats'],'remote':results
_groups_imports['meats']},
    'dairy':{'total':results_groups['dairy'],'local':results_groups_domestic['dairy'],'remote':results_g
roups_imports['dairy']},
 }
# end for year in years
```

Table A. FAO items, codes and the corresponding food group. The codes without parentheses refer to commodity aggregates reported in the food balance sheets, and the codes in parentheses refer to the corresponding items in the "Production" data in the FAOSTAT database [2]. The conversion factors were used to convert quantities to their primary item equivalent (e.g. wine gets converted to equivalent quantity of grapes) [7].

Commodity	FAO codes	Food group	Conversion
Fruits	2919 (1801)	Fruits	-
Wine	2655 (560)	Fruits	0.7
Vegetables	2918 (1735)	Vegetables	-
Starchy Roots	2907 (1720)	Vegetables	-
Cereals	2905 (1717)	Grains	-
Beer	2656 (44)	Grains	4.78
Beverages, Alcoholic	2658 (1717)	Grains	0.6
Bovine meat	2731 (867)	Meat/Protein	-
Mutton and Goat meat	2731 (977,1017)	Meat/Protein	-
Pig meat	2733 (1035)	Meat/Protein	-
Poultry meat	2734 (1058)	Meat/Protein	-
Eggs	2744 (1062)	Meat/Protein	-
Oil crops	2913 (1732)	Meat/Protein	-
Treenuts	2912 (1729)	Meat/Protein	-
Pulses	2911 (1726)	Meat/Protein	-
Milk	2948 (882)	Dairy	-
Butter, Ghee	2740 (886)	Dairy	0.047
Oils	2914 (1732)	Oils	0.2
Sugar crops	2908 (156,157,161)	Discretional	-
Sugar and Sweeteners	2909 (156,157,161)	Discretional	0.12
Stimulants	2922 (661,656)	Discretional	-

Table B. Top livestock product producing countries on average since 1990.

Rank	Cattle meat	Cow milk	Sheep meat	Chicken meat	Pig meat	Hen eggs
1	United States	United States	China	United States	China	China
2	Brazil	Russia	Australia	China	United States	United States
3	China	India	New Zealand	Brazil	Germany	Japan
4	Argentina	Germany	U.K.	Mexico	Spain	India
5	Russia	France	Turkey	Japan	Brazil	Russia
6	Australia	Brazil	Iran	U.K.	France	Mexico
7	France	China	Sudan	Russia	Canada	Brazil
8	Mexico	U.K.	India	France	Poland	France
9	Germany	Ukraine	Spain	India	Netherlands	Germany
10	Canada	New Zealand	Russia	Thailand	Russia	Italy

Fig A (1 - 7) Graphical representation of each continent showing the amount of land spared or required in total to meet the recommendations in the Dietary Guidelines for Americans 2010. The red depicts the amount of land spared or required domestically while the blue line combines domestic land and displaced land to depict a total amount of land spared overall. A negative surplus is to be interpreted as a deficit, meaning that the country would need more food from that group to follow the guidelines.

1) Africa dataset. Graphical representation of Africa showing the amount of land spared or required in total to meet the recommendations in the Dietary Guidelines for Americans 2010. The red depicts the amount of land spared or required domestically while the blue line combines domestic land and displaced land to depict a total amount of land spared overall. A negative surplus is to be interpreted as a deficit, meaning that the country would need more food from that group to follow the guidelines.

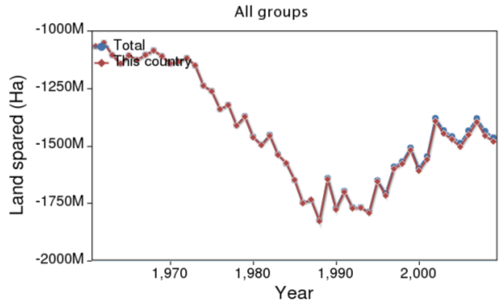

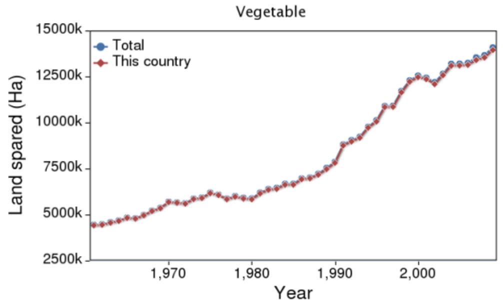

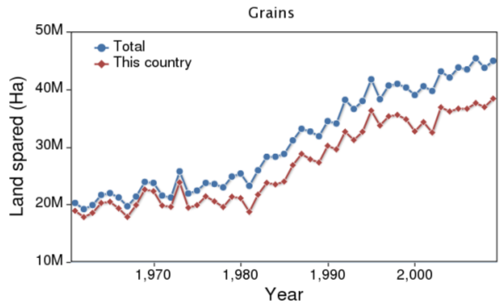

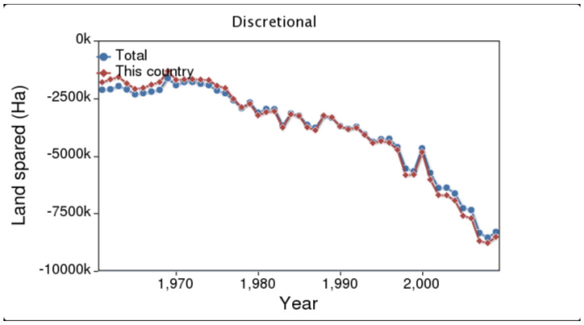

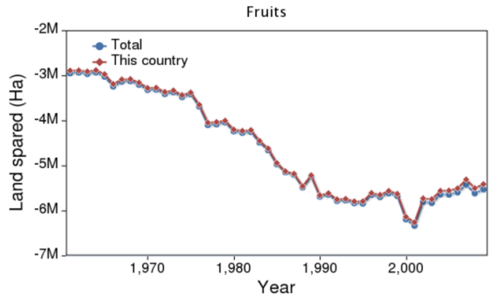

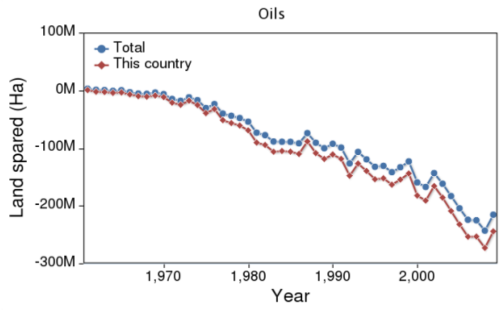
Oils
100M
0M
-100M
-200M
-300M
Total
This country
Land spared (Ha)
1,970
1,980
1,990
2,000
Year

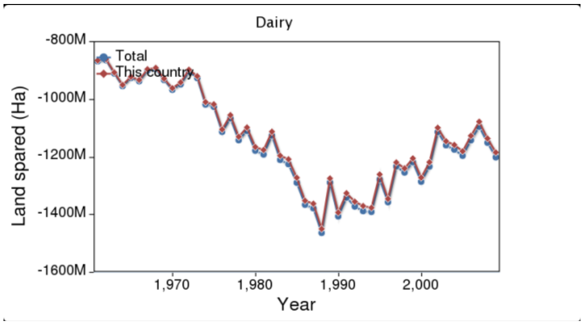
Dairy
-800M
-1000M
-1200M
-1400M
-1600M
Total
This country
Land spared (Ha)
1,970
1,980
1,990
2,000
Year

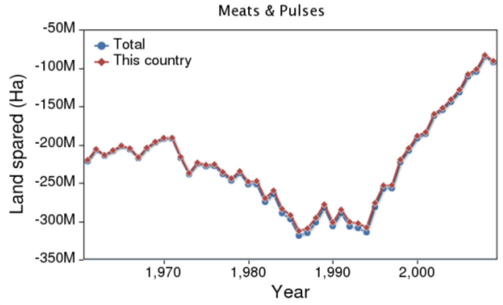
Meats & Pulses
-50M
-100M
-150M
-200M
-250M
-300M
-350M
Total
This country
Land spared (Ha)
1,970
1,980
1,990
2,000
Year

2) Asia dataset. Graphical representation of Asia showing the amount of land spared or required in total to meet the recommendations in the Dietary Guidelines for Americans 2010.

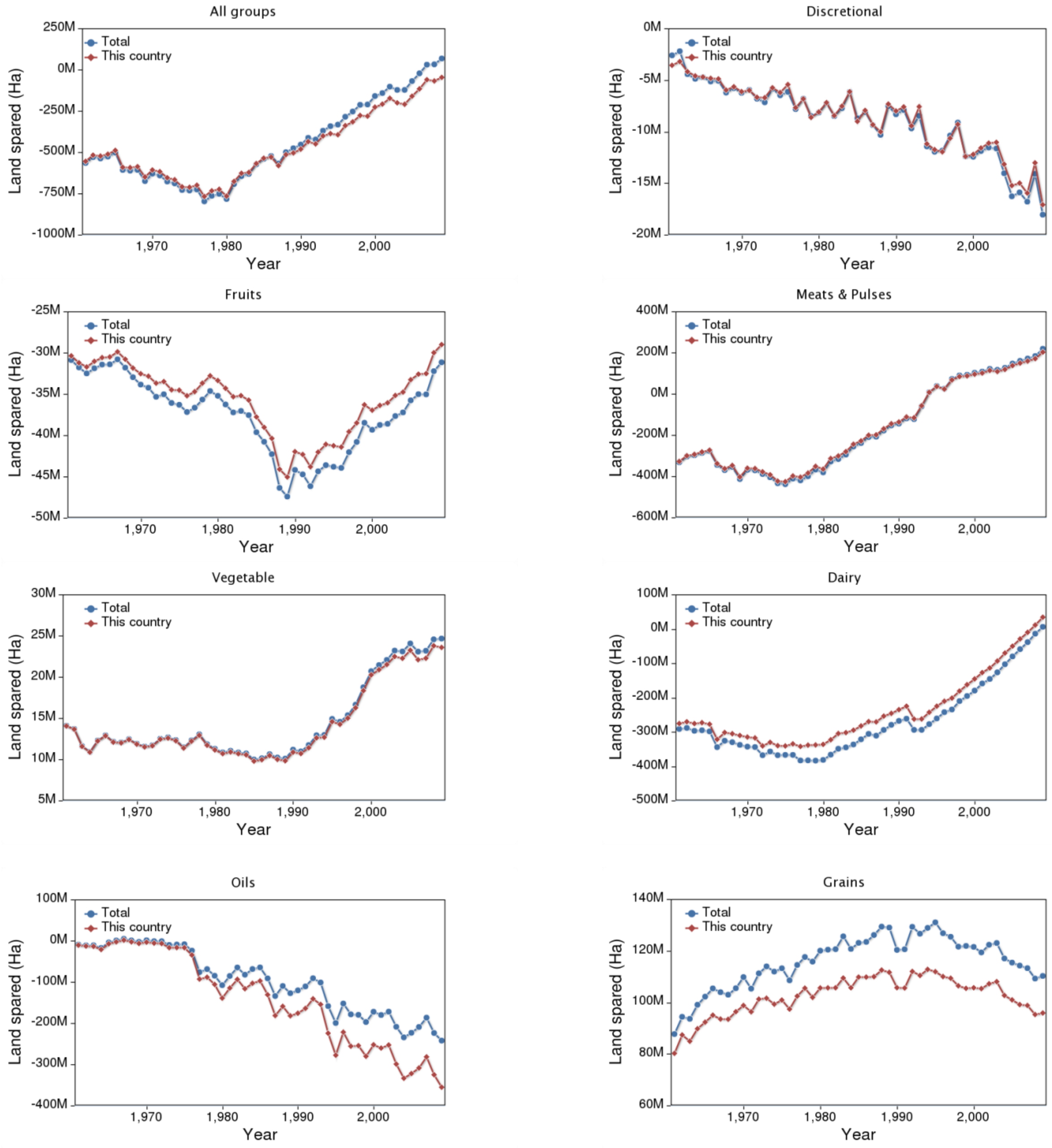

3) Eastern Europe dataset. Graphical representation of Eastern Europe showing the amount of land spared or required in total to meet the recommendations in the Dietary Guidelines for Americans 2010.

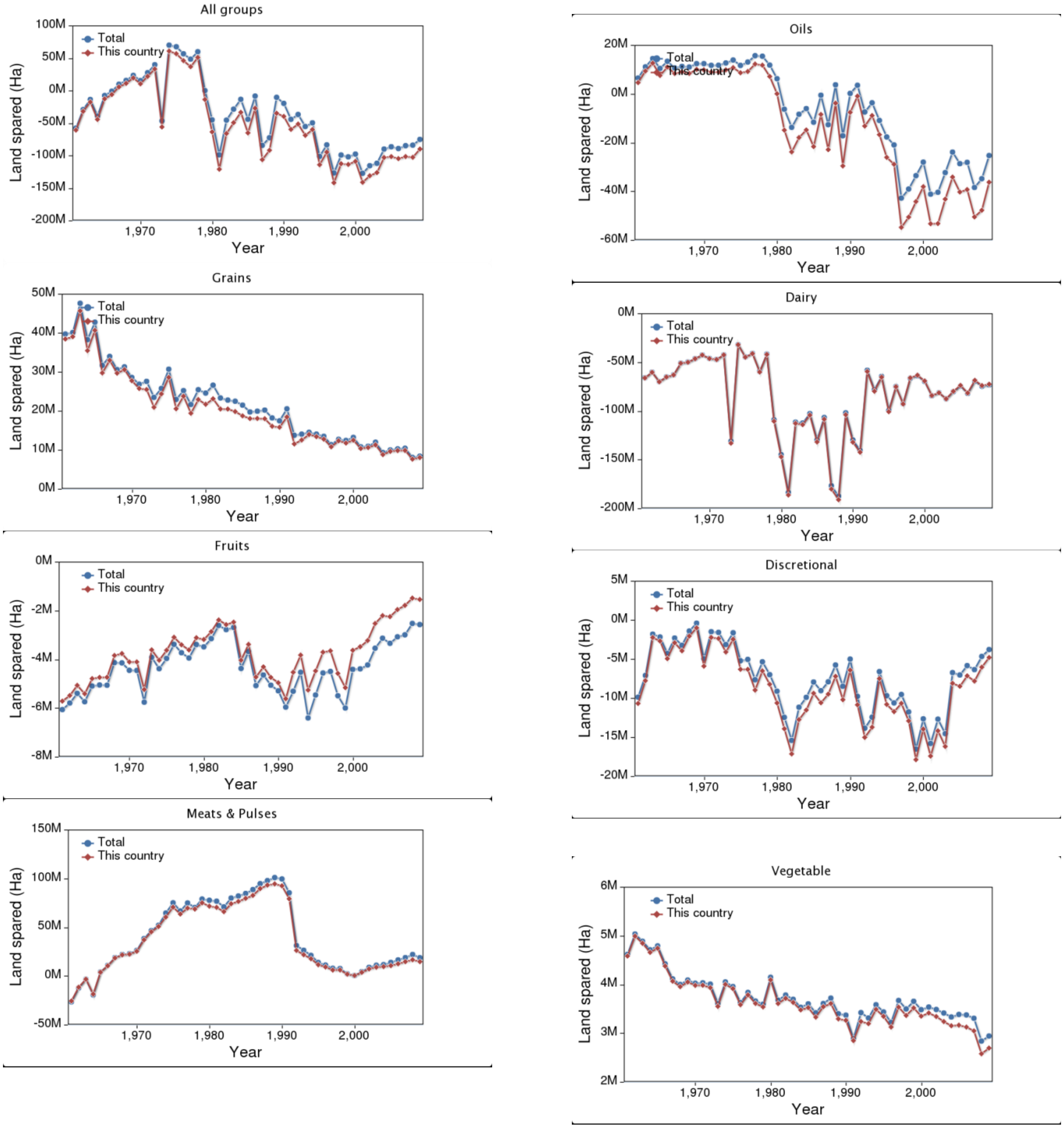

4) European Union dataset. Graphical representation of the European Union showing the amount of land spared or required in total to meet the recommendations in the Dietary Guidelines for Americans 2010.

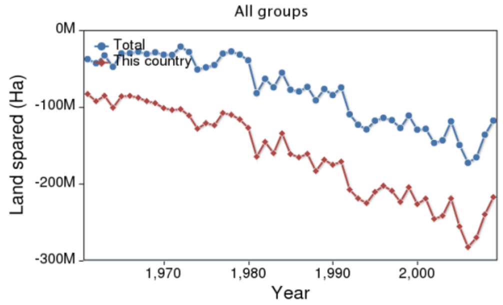

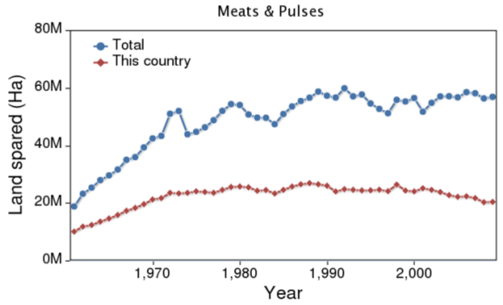

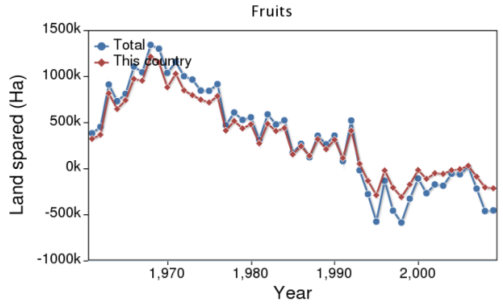

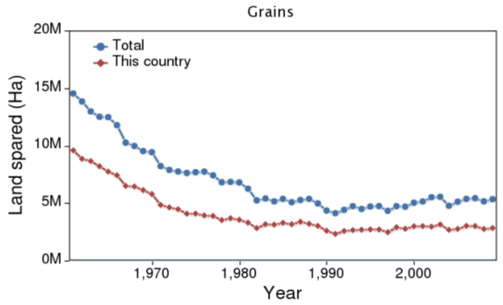

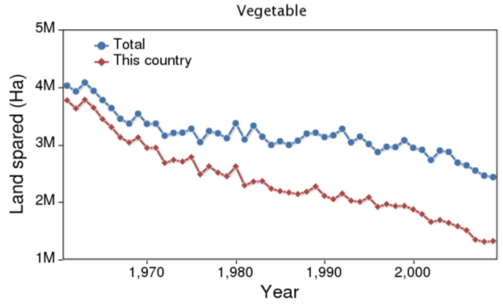

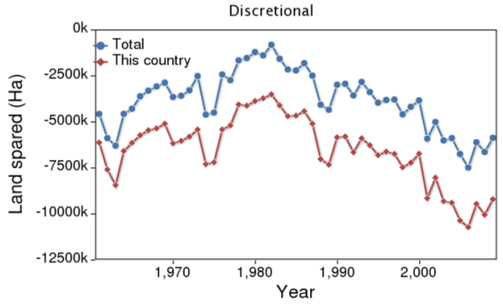

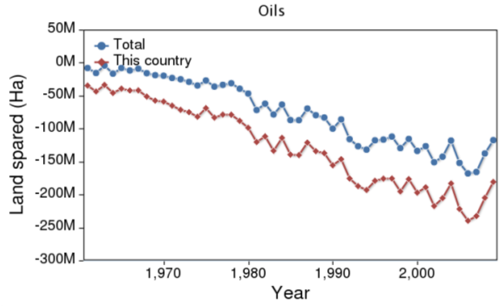

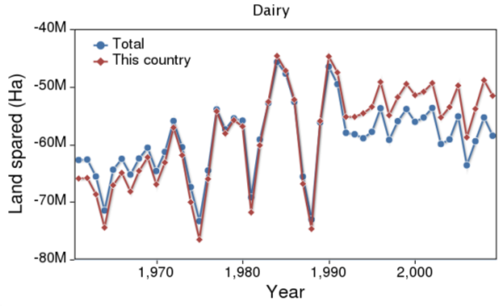

5) North America dataset. Graphical representation of North America showing the amount of land spared or required in total to meet the recommendations in the Dietary Guidelines for Americans 2010.

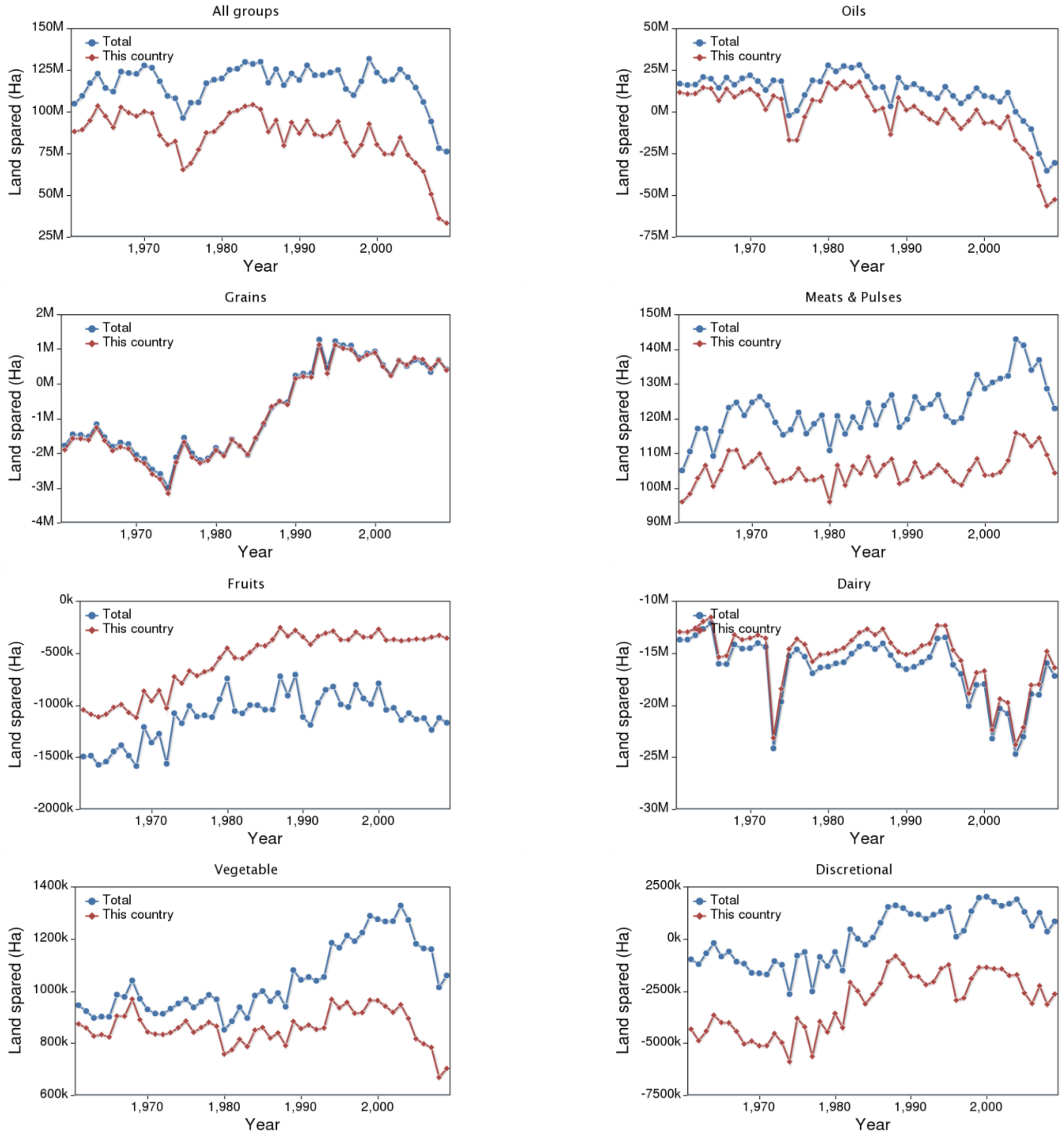

6) Oceania dataset. Graphical representation of Oceania showing the amount of land spared or required in total to meet the recommendations in the Dietary Guidelines for Americans 2010.

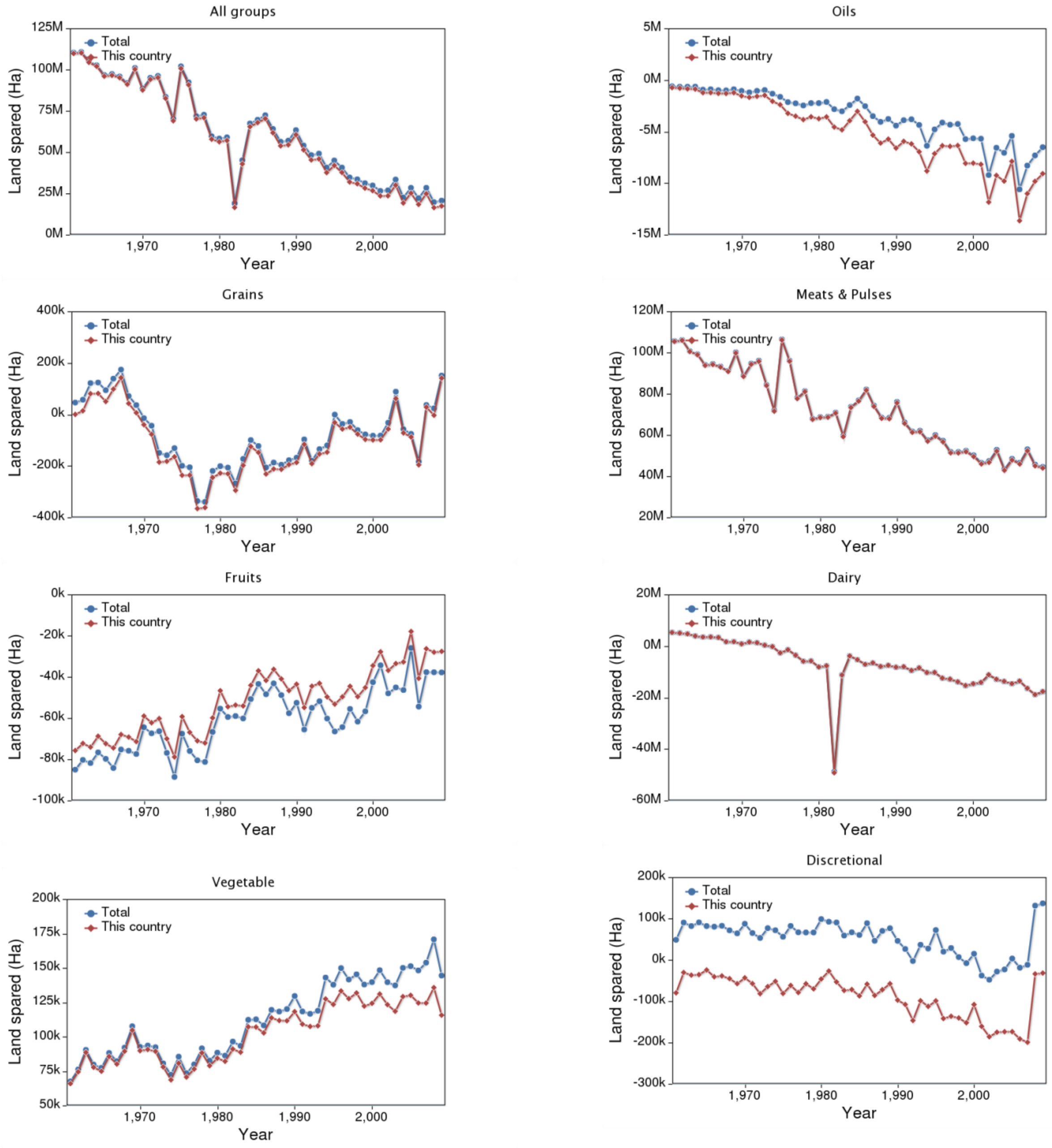

7) South America dataset. Graphical representation of South America showing the amount of land spared or required in total to meet the recommendations in the Dietary Guidelines for Americans 2010.

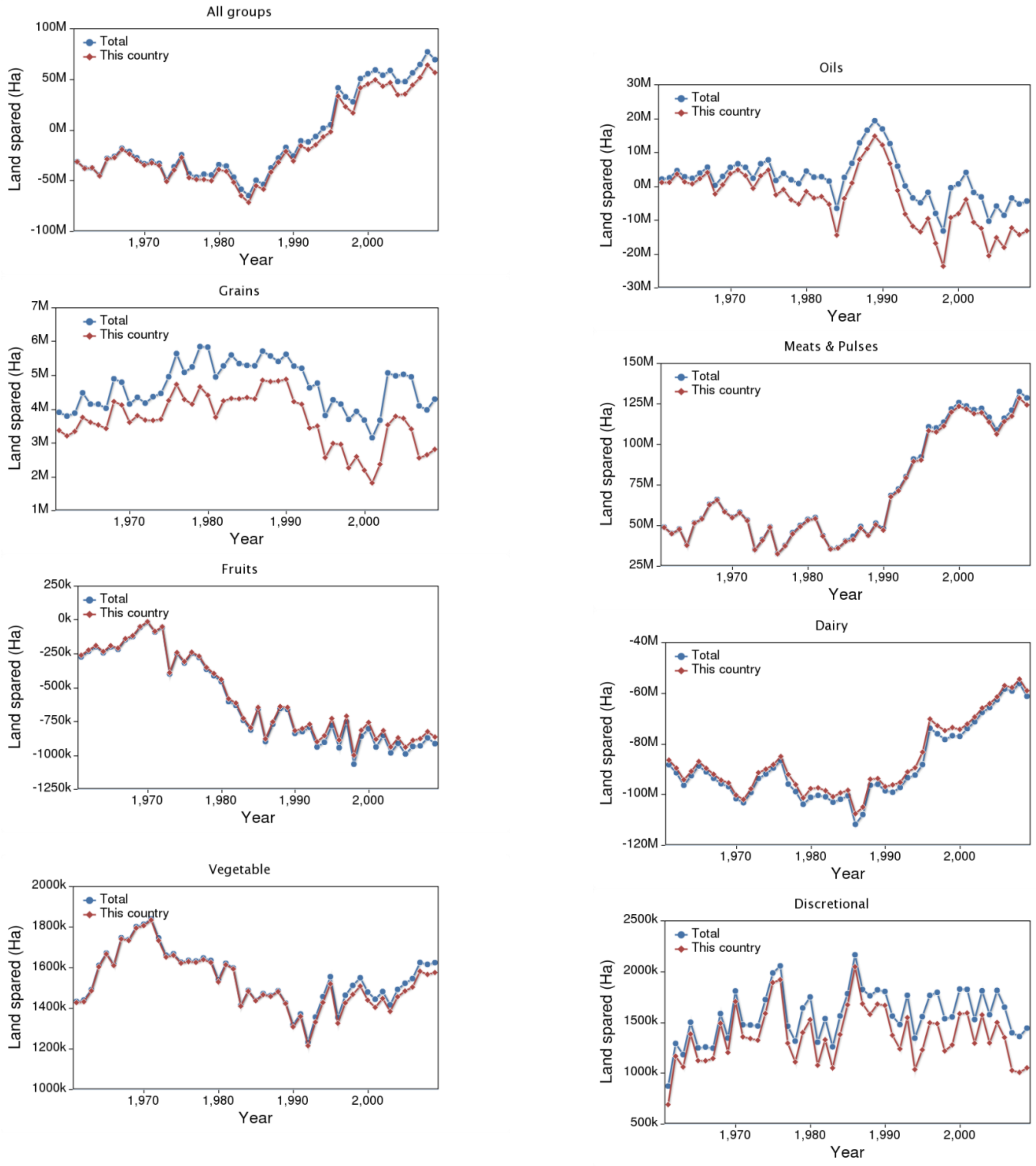

Supplement: S1 Appendix — (PDF) [file pone.0200781.s001.pdf]
